# A Unique, Consistent Identifier for Alternatively Spliced Transcript Variants

**DOI:** 10.1371/journal.pone.0007631

**Published:** 2009-10-28

**Authors:** Alberto Riva, Graziano Pesole

**Affiliations:** 1 Department of Molecular Genetics and Microbiology and University of Florida Genetics Institute, University of Florida, Gainesville, Florida, United States of America; 2 Department of Biochemistry and Molecular Biology “E. Quagliariello”, University of Bari, Bari, Italy; 3 Istituto Tecnologie Biomediche, Consiglio Nazionale delle Ricerche, Bari, Italy; Centre de Regulació Genòmica, Spain

## Abstract

**Background:**

As research into alternative splicing reveals the fundamental importance of this phenomenon in the genome expression of higher organisms, there is an increasing need for a standardized, consistent and unique identifier for alternatively spliced isoforms. Such an identifier would be useful to eliminate ambiguities in references to gene isoforms, and would allow for the reliable comparison of isoforms from different sources (e.g., known genes vs. computational predictions). Commonly used identifiers for gene transcripts prove to be unsuitable for this purpose.

**Methodology:**

We propose an algorithm to compute an isoform signature based on the arrangement of exons and introns in a primary transcript. The isoform signature uniquely identifies a transcript structure, and can therefore be used as a key in databases of alternatively spliced isoforms, or to compare alternative splicing predictions produced by different methods. In this paper we present the algorithm to generate isoform signatures, we provide some examples of its application, and we describe a web-based resource to generate isoform signatures and use them in database searches.

**Conclusions:**

Isoform signatures are simple, so that they can be easily generated and included in publications and databases, but flexible enough to unambiguously represent all possible isoform structures, including information about coding sequence position and variable transcription start and end sites. We believe that the adoption of isoform signatures can help establish a consistent, unambiguous nomenclature for alternative splicing isoforms. The system described in this paper is freely available at http://genome.ufl.edu/genesig/, and supplementary materials can be found at http://genome.ufl.edu/genesig-files/.

## Introduction

Alternative splicing is emerging as a major molecular mechanism to extend the repertoire of functions produced by individual genes, through the expression of multiple transcripts encoding proteins with different biochemical and physical properties, and to diversify the regulation of their expression through alternative 5′ and 3′UTRs. It is now known that the majority of genes in higher organisms are alternatively spliced [1]–[3]. The increasingly important role of alternative splicing in many biological processes [4], [5] and its involvement in various diseases, including cancer [6], [7], has raised enormous interest in further understanding this fundamental process. At the same time, the rapid development of high-throughput RNA sequencing methods now provides a way to observe splicing events directly and on a very large scale, increasing the amount of data available on this phenomenon by many orders of magnitude [1]. This in turn has promoted the development of software algorithms for the computational investigation of alternative splicing, and of specialized databases collecting alternatively spliced gene isoforms from several species with related structural and functional information [2], [8]–[11].

In order to obtain a comprehensive overview of the splicing pattern of a given gene and of its implications, it is in general necessary to consider results obtained by different approaches, both computational and experimental in nature, since no individual method or algorithm is powerful and accurate enough to provide a complete picture of such a complex biological phenomenon. Combining and comparing splicing information from different sources requires the ability to uniquely identify a splicing isoform on the basis of its structure. For example, we may be interested in determining whether a computationally predicted splicing isoform matches an already known one, or one predicted with a different method: accomplishing this by explicitly comparing the arrangements of exons and introns is tedious and error-prone. Using an identifier associated with the exon/intron structure, these tasks would instead reduce to simple identifier comparisons. The ability to uniquely identify a splicing isoform is also useful to eliminate ambiguities when referring to locations within a gene transcript. For example, the PKM2 gene in human encodes for two different isoforms, denoted M1 and M2, characterized by two mutually exclusive exons 9, corresponding to the normal and cancer-specific isoform, respectively [12], [13]. In this situation, a reference to exon 9 is only meaningful if the isoform being considered is also specified, in an unambiguous way.

There have been several efforts aimed at developing a standardized nomenclature for splicing events, and formalisms to describe them. Sammeth *et al.* developed a notation that assigns a unique code to every possible pattern of splicing variation [14]. The code is based on the relative positions of the the splice sites, and can be used to automatically annotate the entire set of alternative splicing events in a set of annotated transcripts. Lee *et al.* proposed the use of *splicing graphs* to represent all possible splicing isoforms generated by the same gene in graphical form [15]. Similarly, Nagasaki *et al.* describe a method to represent and classify every possible alternative splicing configuration using bit vectors [16]. Although these solutions are very useful to classify and describe patterns of splicing events, they do not directly address the problem of identifying the transcripts produced by those events in a general and exact way: there may easily be isoforms produced by the same pattern of splicing events that generate very different transcripts. Similarly, different splicing patterns in different genes can result in isoforms that have exactly the same structure. Finally, these representations are not compact and readable enough to be used as identifiers in publications or in database searches.

We therefore believe there is a need for a standardized and consistent method to uniquely identify an alternatively spliced isoform using a short and unambiguous identifier, whose purpose is to describe its structure, in terms of the exact succession of exons and introns that characterizes it, in a simple and concise way. The identifier, called “isoform signature” in the remainder of this paper, should have the following properties:

1. It should only depend on the lengths and relative positions of the gene exons, and not on their absolute positions on a chromosome (since they may not be known, or may change when the genome is re-assembled), nor on their DNA sequence.
2. It should take into account the fact that the transcription start site (TSS) and the transcription termination site (TTS) cannot always be determined exactly, and should therefore allow identifying isoforms that differ only for the position of the TSS and TTS within a specified range.
3. It should provide a way to optionally include information about the position of the coding sequence, when known.
4. It should not depend on the gene name or other identifier, or on the organism. On the contrary, one of the purposes of the isoform signature is to possibly identify different genes having isoforms with the same structure, possibly belonging to different organisms.
5. It should be short and easy to compute, so that identifiers for newly discovered isoforms can be quickly generated and easily included into publications. It should also have a predictable maximum length, in order to appropriately define the size of fields to contain it in databases.

Existing identifiers for transcripts (such as NCBI's “NM_” accessions or ENSEMBL “ENST” identifiers) are unsuitable for this purpose, for several reasons: they are usually dependent on a specific database, they only apply to known, observed isoforms, and not to computationally predicted ones, and they do not provide a way of detecting when isoforms from different genes in the same or different organisms, or from the same gene with different prediction methods, have the same structure, i.e. the same number and size of exons and introns. More fundamentally, the relationship between genes and transcripts is not bi-univocal: there may be cases in which two paralogous copies of a gene with identical structure produce different transcripts (if their corresponding DNA sequence is different), or cases in which two gene copies, although differing in structure, can produce the same transcript (for example, if the difference consists in one or more introns with different length).

In this paper we propose a method for the generation of an isoform signature that satisfies the above outlined requirements. We describe the algorithm used to compute the signature, providing several examples showing how isoform signatures are calculated, and we present an example of the use of isoform signatures in a large-scale comparison between different databases of alternative splicing information. Finally, we describe a web-based application that we developed that allows users to generate isoform signatures or to search a database of known and predicted gene isoforms using their signatures.

## Results

We define two different types of isoform signature: a *basic* one, that only specifies the exon/intron structure of the isoform, and an *extended* one that also encodes the start and end position of the coding sequence (CDS). Although the CDS position is not technically part of the isoform structure, its biological importance is so high that we provide a way to include it in the signature when it is known. An isoform signature is a short string composed of the following three elements: a 10-character cryptographic identifier, a separator character, and a number. The cryptographic identifier is generated by encoding the arrangement of exons and introns in the isoform as a character string and applying the SHA-1 cryptographic hash function to it, according to the algorithm described in the Methods section. The separator character is a pipe character (“|”) for basic signatures, and a colon character (“:”) for signatures that include the CDS. The number appearing after the separator character is the number of exons in the isoform. The following are examples of isoform signatures: “40a6839e0b|9”, “c58ffac1ac:9”. The first one is a basic signature, the second one includes CDS information.

In the remainder of this paper we show how isoform signatures can be used to identify alternative splicing isoforms without ambiguities, and to detect identities and similarities among different isoforms. While the fact that two isoforms receive the same identifier guarantees that their intron/exon structures are identical, we provide methods to detect isoforms that differ only for the position of the transcription start and end sites, and to take into account the coding sequence position when known.

It is important to note that the isoform signature is a function *only* of the length of the alternating exons and introns, not of their actual position in the genome, nor of their DNA sequence. This is intentional, because the purpose of the signature is to unequivocally identify an isoform *structure*, and to directly compare isoform structures independently of their origin. On the other hand, the signature generation algorithm is extremely sensitive to noise: a change in the position of even a single intron/exon boundary will result in a totally different signature. Biologically, a shift in the start or end position of a coding exon, caused for example by an insertion or by inaccurate splicing, can have dramatic consequences, possibly causing a translation frame shift. In this case, we believe it is appropriate for the isoform signature to change, since the isoforms in question will clearly be distinct. The start and end positions of the transcript are an exception, as noted above: real or predicted isoforms that differ only for their TSS and TTS positions can produce biologically equivalent transcripts. In our approach we handle this issue through the use of *normalized signatures* that link together the signatures of all isoforms whose TSS and TTS lie within a specified range.

A limitation of our proposed method is that, since the SHA-1 algorithm is not invertible, there is no direct way to “decode” an isoform signature and retrieve the corresponding signature string. This can be obviated by creating a database, such as the one described below, associating isoform signatures with the information used to generate them, including the organism name, gene and transcript identifiers.

### The Genesig server

We have developed an online application that implements the isoform signature algorithm described in this paper. The system allows users to easily compute the isoform signature for one or more gene isoforms, and to decode it by searching a local database of precomputed signatures.

The application can operate in two distinct modes: *Generate* and *Lookup*. In Generate mode, it allows the user to compute the signature string, the isoform signature, and the four normalized signatures given an isoform structure. The structure can be specified in one of several different ways:

1. By manually entering the start and end coordinates of the exons or providing them in an uploaded file;
2. By uploading an annotated sequence file, in which the locations of the exons are indicated by a change in case or by special marker characters;
3. By uploading a file in GTF or Aceview format;
4. By entering a signature string.

**Figure 1:**
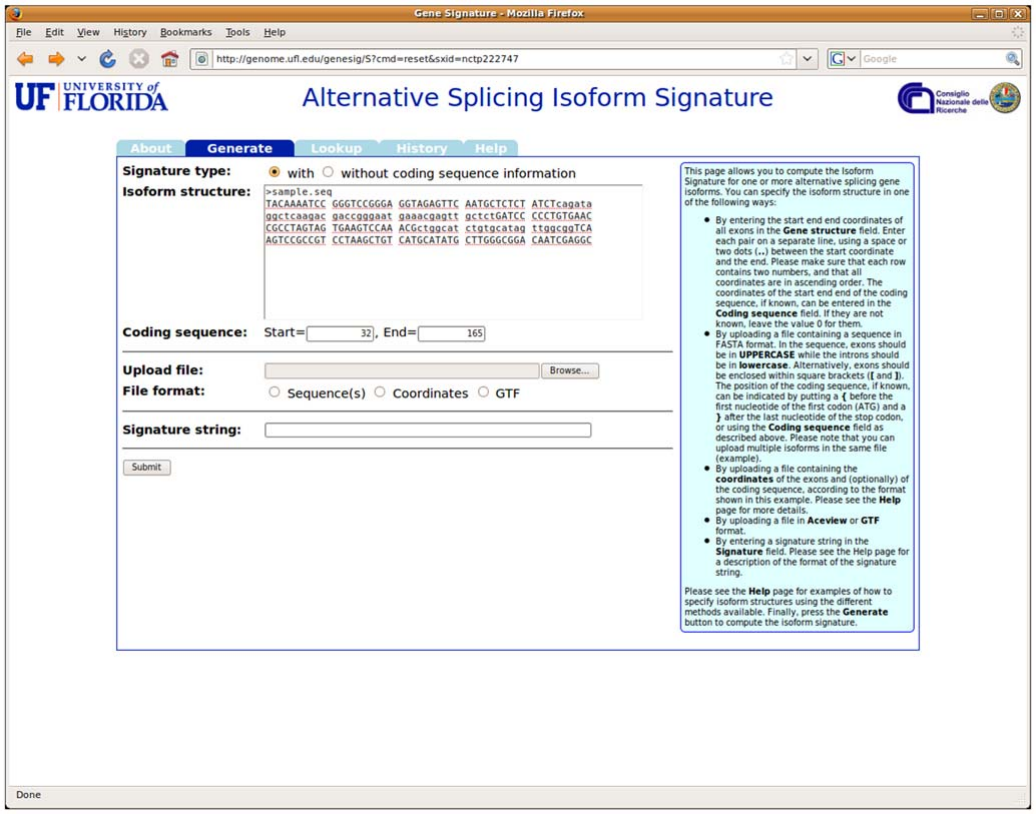
The input form for the *Generate* section of the website. The user may specify the isoform structure(s) in a variety of different ways, including pasting a specially-formatted FASTA sequence or uploading it from a file.

**Figure 2:**
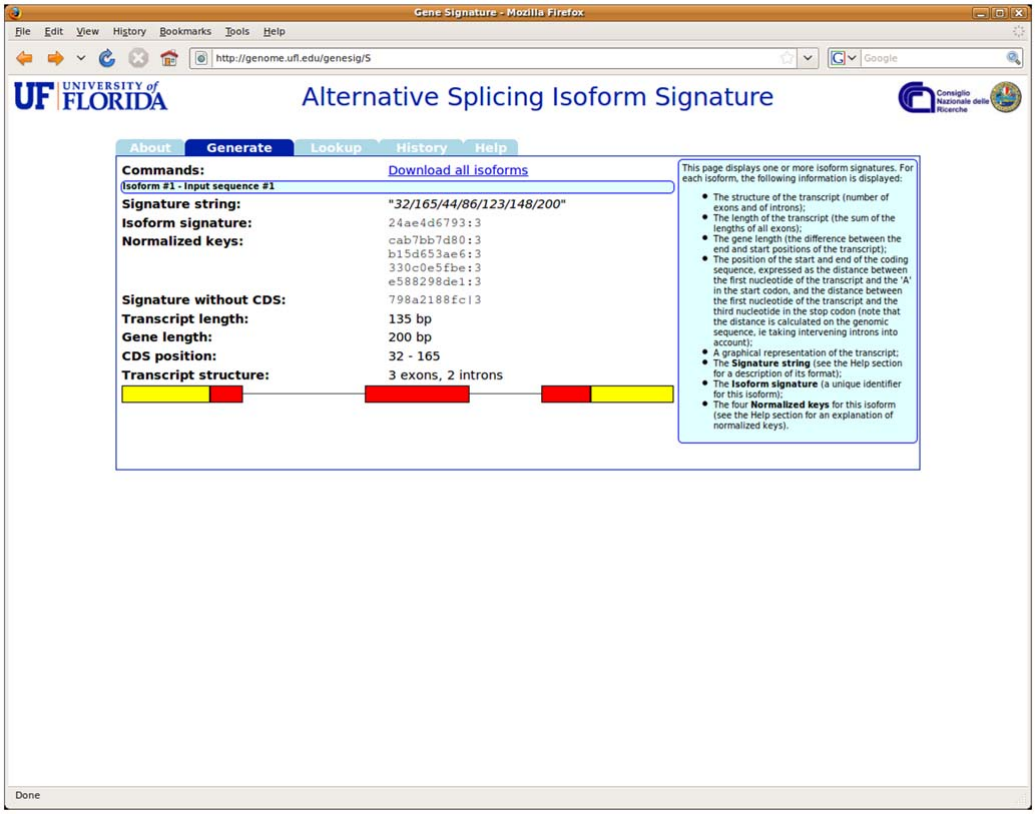
The output of the *Generate* command. For each supplied isoform, the system displays the signature string, the main and alternative isoform signatures, and details of the isoform structure including its graphical representation.

In all cases, the user has the choice of generating an extended isoform, by providing coding sequence position information, or a basic one (see Figure 1). The application decodes the isoform structure (or structures) described by the user's input, generates the corresponding signatures, and displays them on the resulting page (see Figure 2). It should be noted that the system can generate signatures for multiple isoforms at once, when the input comes from an uploaded file. Each signature is a link to the Lookup section of the application, described below: the user can thus quickly verify whether a generated signature corresponds to a known isoform. The results can also be downloaded to a text file, so that they can easily be inserted in databases or used for further processing.

**Figure 3:**
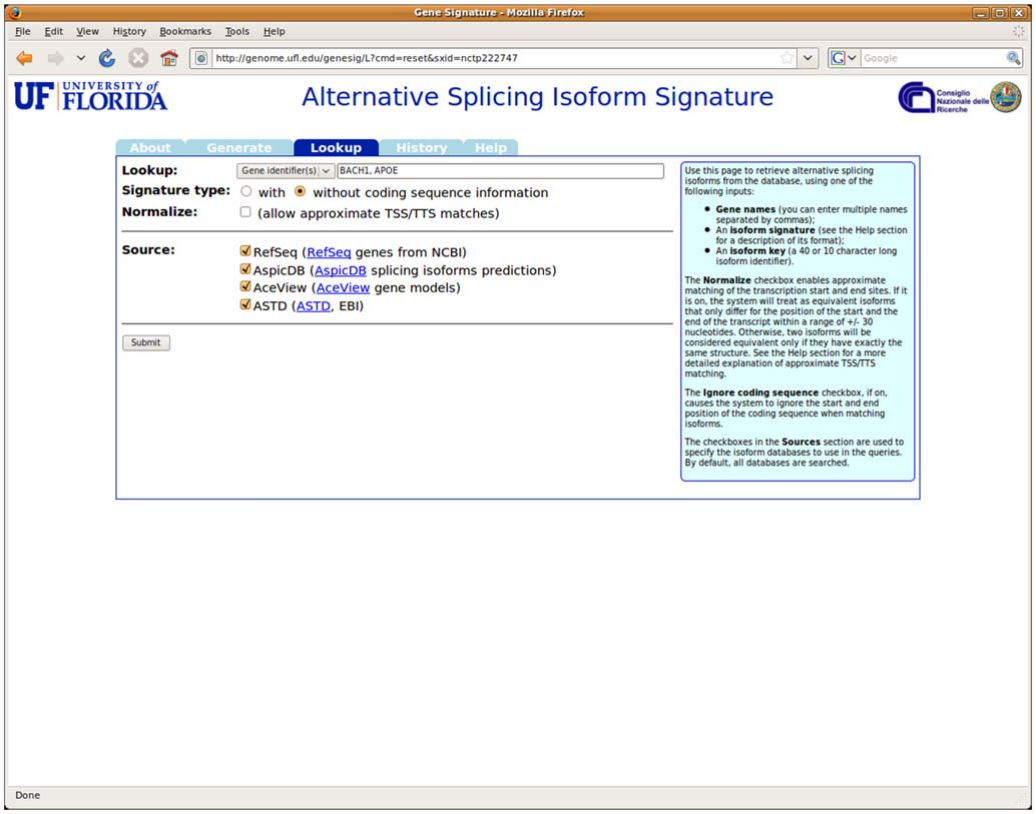
The input form for the *Lookup* section of the website. The user may enter an isoform signature, a signature string, or a list of gene names as the query term. It is also possible to specify what kind of search to perform (exact or approximate, with or without coding sequence information) and the list of isoform databases to search.

**Figure 4:**
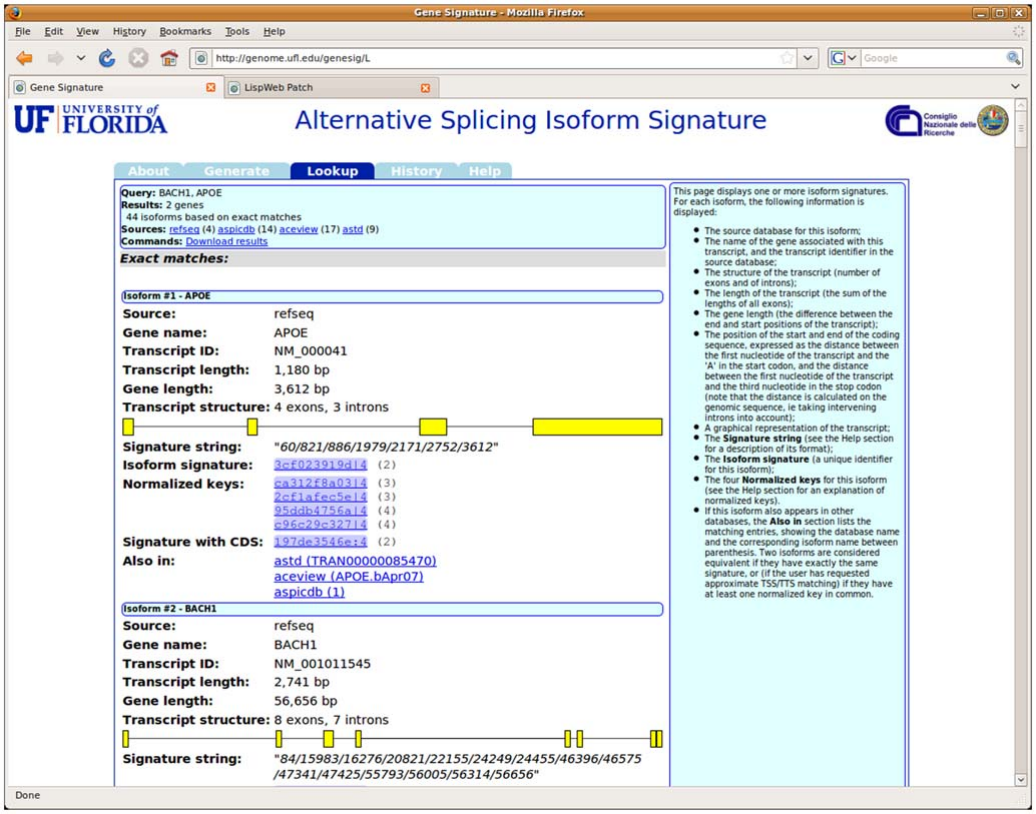
The output of the *Lookup* command. The system displays the list of retrieved isoforms matching the supplied query terms, providing information about their signatures and their structures. All the results displayed in this page can be downloaded to a local file.

In Lookup mode, the application takes as input an isoform signature, a signature string, or a set of gene names, and retrieves matching isoforms from its database. The user may optionally specify whether to perform an “approximate” search (one that uses the normalized signatures in addition to the main one) and whether to take into account the position of the coding sequence or ignore it (see Figure 3). Finally, the user may restrict the search to a subset of the sources of isoform data included in the application's database (all of them are used by default). The current version of the system includes isoform signatures for all transcripts contained in the following databases: NCBI's Refseq, AspicDB [2], Aceview [10] and ASTD [9]. When a lookup is successful, the system displays all matching isoforms found in the database providing details about the source database they appear in, the gene they belong to, and the structure of isoform, including a graphical display of the exon/intron arrangement (see Figure 4). As in the previous case, all results can be downloaded as a delimited text file. The Lookup function can also be invoked using a specially formatted URL that includes the signature: when included in a web page, this URL will produce a link leading to the page displaying the information about the corresponding isoform.

**Figure 5:**
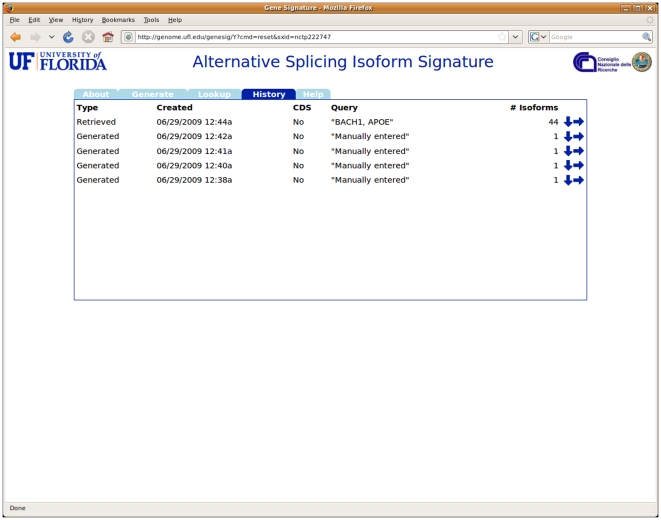
The History section lists all sets of isoforms generated or retrieved during the current session, and provides links to display their contents or to download them to local files.

Finally, a *History* feature allows the user to list all the isoform signatures generated or retrieved in the course of the current session (see Figure 5). The user can choose to display each individual set of signatures or to download the signature data to a local file.

The application is web-based and can be freely accessed at the URL http://genome.ufl.edu/genesig/. Extensive documentation about the signature generation method and detailed help pages to facilitate using the application are provided.

### Application

We performed a large-scale comparison of the computationally predicted alternative splicing isoforms of human genes contained in three different databases: ASPICdb [2], ASTD [9], and ACEVIEW [10]. Since these predictions were generated using different methodologies, we were interested in evaluating the level of agreement among them, as well as between each one of them and the NCBI Refseq database. Refseq provides a comprehensive, non-redundant and well-annotated set of transcript sequences for several organisms, including several splicing isoforms (Refseq release 32, of November 2008, contains 38,827 transcripts from 17,787 multi-exon human genes), and can therefore be used to provide an estimate of the quality of predicted splicing isoforms.

The comparison was performed by generating the signature for every predicted transcript, and calculating the percentage of matching isoforms for corresponding genes in each pair of databases. We considered both exact matches and approximate matches (those based on the alternative signatures), with or without taking CDS information into account. Results are shown in Table 1. ASPICdb exhibited the best performance in terms of matching transcript signatures: using the approximate method, 91% of Refseq transcripts were correctly matched, with 93% of genes showing at least one matching signature. Excluding the CDS annotation from the signature definition, we obtained a slight increase in the percentages of matching signatures (92% and 94%, respectively). The percentage of matching isoform signatures between Refseq and the two other databases was much lower, ranging from 36% (ACEVIEW) to only 10% (ASTD), in the same conditions. Concerning the pairwise comparisons between ASPICdb, ACEVIEW and ASTD, the number of matching transcript signatures resulted generally low, ranging from 25% (ACEVIEW vs ASTD) to 14% (ASPICdb vs ASTD), when using the approximate method and without considering the CDS (Table 2).

**Table 1:** Pairwise comparisons between three alternative splicing databases (ACEVIEW, ASTD, and ASPICdb) and RefSeq.

	ACEVIEW	ASTD	ASPICdb	RefSeq
**ACEVIEW**	-	797/945	1691/2175	1531/1919
		(6%/1%)	(13%/2%)	(8%/10%)
**ASTD**	3063/4520	-	1503/1692	519/548
	(24%/6%)		(12%/2%)	(4%/3%)
**ASPICdb**	7874/14070	3983/5626	-	9262/12809
	(62%/11%)	(31%/7%)		(62%/58%)
**RefSeq**	6514/8467	1866/2112	13848/19875	-
	(41%/36%)	(14%/10%)	(93%/91%)	

In each cell, the first figure is the number of genes in common between the two databases (i.e., genes with at least one matching isoform), while the second figure is the number of matching isoforms. The corresponding percentages are reported below each pair of numbers. Only transcript variants from genes present in both compared databases were considered. The position of the CDS was taken into account when comparing isoforms in this analysis.

**Table 2:** Pairwise comparisons between three alternative splicing databases (ACEVIEW, ASTD, and ASPICdb) and RefSeq.

	ACEVIEW	ASTD	ASPICdb	RefSeq
**ACEVIEW**	-	3043/4039	2725/3584	1963/2498
		(24%/5%)	(21%/3%)	(12%/11%)
**ASTD**	7855/18159	-	2525/2979	822/862
	(62%/25%)		(20%/4%)	(6%/4%)
**ASPICdb**	10020/23407	6281/10544	-	9377/12968
	(79%/19%)	(49%/14%)		(63%/59%)
**RefSeq**	7960/10637	2945/3364	14086/20265	-
	(50%/45%)	(22%/17%)	(94%/92%)	

In each cell, the first figure is the number of genes in common between the two databases (i.e., genes with at least one matching isoform), while the second figure is the number of matching isoforms. The corresponding percentages are reported below each pair of numbers. Only transcript variants from genes present in both compared databases were considered. The position of the CDS was not taken into account when comparing isoforms in this analysis.

The results obtained suggest a generally low degree of overlapping between alternatively spliced transcripts collected in different databases as well as between these and RefSeq transcripts, with the notable exception of ASPICdb. Assuming that RefSeq transcripts represent the “gold standard”, the reliability of alternative isoforms predicted by ASPICdb is clearly higher than that of those found in ACEVIEW and ASTD. The better performance of ASPICdb can be partially explained by the fact that, while ACEVIEW and ASTD only use available EST/transcript data, ASPICdb uses EST/transcript data to generate the full set of introns found in a given gene region, and applies a combinatorial assembly algorithm to obtain all compatible full-length isoforms [17]. Furthermore, different exon-intron structures may be due to the use of different alignment algorithms [18]. For example, in the case of the *SLC22A7* gene transcript NM_153320, a 6 bp microexon is detected by ASPICdb (but also appears in the highly curated annotations provided by VEGA [19], see entry OTTHUMT00000040588) but not by RefSeq, ACEVIEW or ASTD. Therefore, ASPICdb shows a transcript with 11 exons, while those in the other databases only have 10. These deviations can be very easily detected using our system, since they will result in different isoform signatures. Finally, ASPICdb predictions are based on data that are more up-to-date than those in ACEVIEW and ASTD.

The Supplementary Materials site provides downloadable files containing signatures for all gene isoforms in the four databases considered in this study.

## Discussion

As demonstrated by the cross-database comparison we performed, different computational methods for the prediction of splicing isoforms vary greatly in the number of predictions they produce, in the concordance of their predictions with known, manually validated isoforms, and in the level of agreement with other similar resources. As our knowledge of the causes and potential biological and clinical consequences of alternative splicing grows, it will become increasingly important to have a way of uniquely identifying splicing isoforms, so that information like exon numbering and relative positions of genetic elements within transcripts can be determined without ambiguity.

In this work we proposed a simple and efficient method for the generation of isoform signatures that uniquely identify an alternative splicing isoform. The method employs the well-known SHA-1 cryptographic hashing algorithm, for which numerous implementations exist in most common programming languages, and can therefore be easily reimplemented. The resulting identifiers are designed to be easily included in publications, databases and existing informational resources. Isoform signatures are a function of the exact arrangement of exons and introns in a transcript, such that a change of even a single base in the length of one of these elements guarantees an entirely different identifier. Nevertheless, our method takes into account the fact that the transcription start and end sites of a transcript cannot always be determined exactly, and may span across few neighboring positions. The use of normalized signatures allows matching of isoforms that differ only in the exact positions of the TSS and TTS within a predefined range. For example, the RefSeq database contains three different isoforms for the human *KLK5* gene. While ASPICdb identifies all three isoforms exactly, ACEVIEW and ASTD each contain only one isoform that matches one in RefSeq exactly (a different one in each case), and two isoforms that match the remaining two in RefSeq except for small changes in the TSS and TTS, of 23 nucleotides at most. These comparisons of different isoforms can be performed very easily using the method we propose, by simply matching isoform signatures or the corresponding signature strings.

We have developed a publicly-available, web-based tool to facilitate the adoption and use of isoform signatures. The system allows users to compute isoform signatures for one or more alternative splicing isoforms at the same time, offering a variety of different methods to describe the isoform structure, and provides its results as a web page or as a downloadable delimited file. The same tool can also be used to look up isoform signatures in a local database containing all known and computationally predicted isoforms for all human and mouse genes (other organisms will be added over time). In this way the user can associate an isoform signature with the corresponding alternative splicing isoform and obtain detailed information about it, including the gene that it belongs to, its exact structure, and its presence in different databases. The method we described is in the public domain, and we encourage publication authors and database developers to adopt isoform signatures in their works, in order to reduce ambiguities in the identification of alternatively spliced isoforms.

## Methods

The following is the procedure we propose to compute the signature of an alternative splicing isoform.

1. Consider a primary transcript sequence composed of alternating exons and introns. The sequence begins with the first nucleotide of the first exon, and ends with the last nucleotide of the last exon. We label each nucleotide with a progressive number starting at 1. The label for the last nucleotide of the sequence will be equal to the length of the sequence.
2. Collect the labels of the initial and final nucleotides of each exon, in ascending numerical order (regardless of the strand that the transcript is on). The first number in this sequence will always be 1 by definition.
3. Create a *signature string* containing all the labels collected in step 2 except for the initial one (that is not necessary since it will always be 1 by definition). The numbers should be written in the string as decimal values separated by a forward slash (‘/’). There should be no slash at the beginning or at the end of the string.
4. Compute the SHA-1 cryptographic hash of this string [20]. The result is a sequence of 160 bits that can be written as a string of 40 hexadecimal digits (padding it with 0 to the left if necessary). The *isoform signature* consists of the first 10 hexadecimal digits (corresponding to the first 40 bits), followed by a pipe character (‘|’), and by the number of exons in the isoform.

If the position of the coding sequence is known, the last two steps of the algorithm are modified in the following way:

- Collect the positions of the start of the coding sequence (the label of the ‘A’ nucleotide in the ATG) and of the end of the coding sequence (the label of the third nucleotide in the stop codon). Create a signature string containing the start and end of the coding sequence followed by all the labels collected in step 2 except for the initial one (that is not necessary since it will always be 1 by definition). The numbers should be written in the string as decimal values separated by a forward slash (‘/’). There should be no slash at the beginning or at the end of the string.
- Compute the SHA-1 cryptographic hash of this string. The result is a sequence of 160 bits that can be written as a string of 40 hexadecimal digits (padding it with 0 to the left if necessary). The *isoform signature* consists of the first 10 hexadecimal digits (corresponding to the first 40 bits), followed by a colon character (‘:’), and by the number of exons in the isoform.

To account for the fact that, as described above, the TSS and TTS are not always exactly determined, and may span over a limited genomic region, the algorithm is extended to generate an additional set of “normalized” keys associated with each isoform. Normalized keys are produced by forcing the lengths of the first and last exons of the isoform to a fixed set of values. As will be shown below, isoforms that differ only for the length of their initial and/or final exons will share at least one of their normalized signatures, and this fact can be used to establish a relationship between them.

The following additional steps of the algorithm are used to compute the four alternative signatures:

1. Choose an appropriate range of variability for the TSS (called R_S_) and a similar range of variability for the TTS (R_T_). In this work we used R_S_ = R_T_ = 30 nt.
2. Let L_S_ be the length of the first exon in the transcript. Determine the multiple of R_S_ immediately preceding L_S_ and the one immediately after it, and call them P_S_ and Q_S_. For example, if L_S_ is 213 nt and R_S_ is 30, we obtain P_S_ = 210 and Q_S_ = 240.
3. Let L_T_ be the length of the final exon in the transcript. Determine the multiple of R_T_ immediately preceding L_S_ and the one immediately after it, as in the previous step, calling them P_T_ and Q_T_ respectively.
4. Modify the start position of the first exon so that its length becomes equal to P_S_, and the end position of the last exon so that its length becomes equal to P_T_. Apply steps 3 and 4 above to the resulting set of coordinates to generate an *alternative signature*. Repeat using Q_S_ and P_T_, P_S_ and Q_T_, Q_S_ and Q_T_. The result is a set of four different alternative signatures that are associated with the primary signature obtained in the first part of the algorithm.

This procedure ensures that if two gene isoforms differ only for the position of the TSS (or of the TTS, or both) and the difference between the positions is less than twice R_S_ (or R_T_, respectively) then their signatures will be different, but they will have at least one of their four alternative signatures in common. See the next section for an example. The value of 30 nt for R_S_ and R_T_ was chosen empirically, observing the normal range of variation in the start and end positions of transcripts, and looking for a tradeoff between a more stringent definition of the isoform structures and the requirement to recognize similar isoforms.

Although the isoform signature only contains one quarter of the total number of bits produced by the cryptographic hash function, it is still sufficient to represent over 10^12^ different values, several orders of magnitude more than the number of known and predicted transcripts in a genome. While in theory there is a chance that two different transcripts could generate the same isoform signature (a collision), in practice the probability of this event is extremely small, and is further reduced by the addition of the number of exons at the end of the signature: a collision can therefore only happen if two different isoforms, *having the same number of exons*, produce hash values whose first 40 bits are identical. We empirically evaluated the likelihood of this occurrence by generating 5 million random isoforms, with a number of exons ranging from 2 to 100, and comparing the signatures generated by them. We repeated this test 5 times, never observing a single collision. The results of this test are available in the Supplementary Materials section. Moreover, we generated isoform signatures for all gene isoforms in the human and mouse genome, including both known and computationally predicted ones. Again, there was no collision between different isoforms in this database.

### Examples

#### Example 1

Consider the following hypothetical primary transcript sequence, in which exons are indicated in uppercase and introns in lowercase:

1234567890 1234567890 1234567890 1234567890 1234567890

1 TACAAAATCC GGGTCCGGGA GGTAGAGTTC AATGCTCTCT ATCTgtgata

51 ggctcaagac gaccgggaat gaaacgagtt gctagGATCC CCCTGTGAAC

101 CGCCTAGTAG TGAAGTCCAA ACGgtggcat ctgtgcatag ttggcagTCA

151 AGTCCGCCGT CCTAAGCTGT CATGCATATG CTTGGGCGGA CAATCGAGGC

The three exons are at positions 1 - 44, 86 - 133, and 148 - 200. The signature string therefore is:

“44/86/133/148/200”

Applying the SHA1 algorithm to this string and converting the result to hexadecimal characters, we obtain the following string:

“2f3cac598c9788009f5096c8185643cad7fdee8c”

And the isoform signature is therefore:

“2f3cac598c|3”

#### Example 2

The mRNA of human gene *APOE* (NM_000041) is composed of 4 exons, encoded at the following positions on chromosome 19 (according to NCBI build 36):

StartEndLength

50,100,87950,100,93860

50,101,69950,101,76466

50,102,85750,103,049993

50,103,63050,104,490861

The coding sequence starts at position 50,101,721 and ends at position 50,104,347. The signature string is therefore:

“844/3469/60/821/886/1979/2171/2752/3612”

where all positions are expressed relative to the start of the transcript (50,100,879) and the first two numbers represent the start and end of the coding sequence. The hexadecimal string produced by the SHA-1 algorithm is:

“197de3546e9d2e134120abb6038ccbdaef3a0292”

And the isoform signature (in this case, an extended signature) is:

“197de3546e:4”

To calculate the alternative signatures for this transcript, let us first determine the length of the initial and final exons. In this case, L_S_ = 60 and L_T_ = 861. Therefore, P_S_ = 30, Q_S_ = 90, P_T_ = 840 and Q_T_ = 870. Note that, since in this example the length of the first exon is already an exact multiple of 30, the previous and next multiples are used. We therefore modify the coordinates of the start of the first exon and of the end of the last exon (corresponding to the start and the end of the transcript respectively) so that the length of the first exon becomes 30 and the length of the last exon becomes 840. The first set of new coordinates is then the following:

StartEndLength

**50,100,909**50,100,938**30**

50,101,69950,101,76466

50,102,85750,103,049993

50,103,630**50,104,469** **840**

The modified coordinates are shown in bold face. Note that this isoform, in general, will not be a biologically “real” one, but is only generated to compute the first alternative isoform signature, which,. in this case, will be the following:

“a4b5a4e342:4”

Any other isoform having the same exact structure as this one, with a first exon whose length is between 1 and 60 and a last exon whose length is between 810 and 870, will have a normalized signature identical to this one (although its main signature will be different), because the lengths of its first and last exons will become 30 and 840, respectively, by rounding them up or down appropriately. The other three alternative signature strings are calculated in a similar way, by setting the lengths of the first and last exons to 90 and 840, 30 and 870, 90 and 870 respectively.
